# An Immune‐Enhancing Injectable Hydrogel Loaded with Esketamine and DDP Promotes Painless Immunochemotherapy to Inhibit Breast Cancer Growth

**DOI:** 10.1002/adhm.202401373

**Published:** 2024-08-09

**Authors:** Xiali Yin, Yaohua Ke, Ying Liang, Shuxian Zhang, Ziqi Chen, Lixia Yu, Ming Jiang, Qin Liu, Xiaoping Gu

**Affiliations:** ^1^ Department of Anesthesiology Nanjing Drum Tower Hospital Clinical College of Nanjing Medical School Nanjing 210008 China; ^2^ The Comprehensive Cancer Centre of Nanjing Drum Tower Hospital The Affiliated Hospital of Nanjing University Medical School & Clinical Cancer Institute of Nanjing University Nanjing 210008 China; ^3^ Department of Anesthesiology Nanjing Drum Tower Hospital The Affiliated Hospital of Nanjing University Medical School Nanjing 210008 China; ^4^ The Comprehensive Cancer Centre China Pharmaceutical University Nanjing Drum Tower Hospital 321 Zhongshan Road Nanjing 210008 China

**Keywords:** breast cancer, esketamine, injectable hydrogel, tumor microenvironment, local immune

## Abstract

Chemotherapy is the cornerstone of triple‐negative breast cancer. The poor effectiveness and severe neuropathic pain caused by it have a significant impact on the immune system. Studies confirmed that immune cells in the tumor microenvironment (TME), have critical roles in tumor immune regulation and prognosis. In this study, it is revealed that the painless administration of Esketamine, combined with Cisplatin (DDP), can exert an anti‐tumor effect, which is further boosted by the hydrogel delivery system. It is also discovered that Esketamine combined with DDP co‐loaded in Poloxamer Hydrogel (PDEH) induces local immunity by increasing mature Dendritic Cells (mDCs) and activated T cells in PDEH group while the regulatory T cells (Tregs) known as CD4^+^CD25^+^FoxP3^+^decreased significantly. Finally, , CD8^+^ and CD4^+^ T cells in the spleen exhibited a significant increase, suggesting a lasting immune impact of PDEH. This study proposes that Esketamine can serve as a painless immune modulator, enhancing an anti‐tumor effect while co‐loaded in poloxamer hydrogel with DDP. Along with improving immune cells in the microenvironment, it can potentially alleviate anxiety and depression. With its outstanding bio‐safety profile, it offers promising new possibilities for painless clinical therapy.

## Graphical abstract

The analgesics currently employed in tumor are immunosuppressive. A painless administration of Esketamine combined with Cisplatin (DDP)co‐loaded in a thermo‐sensitive injectable hydrogel (PDEH) is designed to improve the immunosuppressive tumor microenvironment (TME) by activating Dendritic Cells (DCs), interferon‐γ (IFN‐γ) cells and inhibiting regulatory T cells (Tregs) on murine breast cancer. Furthermore, the anxiety and depression of mice are also relieved.

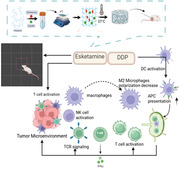

## 1 Introduction

Tumors are one of the major causes of human death. ≈18 million new malignant cases were recorded across the globe in 2021. Breast cancer has been the most prevalent form of tumors in women globally, with a steady annual increase.^[^ ^1^, ^2^ ^]^ Surgery is found to be effective only in the early stages, according to research on the relationship between staging, classification, and cancer prognosis. Chemotherapy is the mainstay of breast cancer treatment, including preoperative, postoperative, and advanced cases.^[^ ^3^, ^4^ ^]^ However, due to its poor targeting, chemotherapy destroys both tumor cells and normal cells. The diminished immune response and heightened immune evasion caused the low efficacy.^[^ ^5^ ^]^ Studies have confirmed that solid tumors have a special microenvironment, composed of extracellular matrices, large numbers of immunosuppressive cells, and immune cells, such as Dendritic cells (DCs), T lymphocytes, natural kill cells (NK), and macrophages.^[^ ^6^, ^7^ ^]^ The immunosuppressive microenvironment severely hampers the efficacy in breast cancer.^[^ ^8^, ^9^ ^]^ Immunotherapy targeting the tumor microenvironment (TME) emerged as the primary option among the indications approved by the Food and Drug Administration (FDA).^[^ ^10^, ^11^ ^]^ Scientific studies have confirmed the crucial role of activated DCs, cytotoxic T lymphocytes (CTLs), and macrophages in exerting an anti‐tumor effect within the microenvironment.^[^ ^12^, ^13^ ^]^ Immune agonists, cytokines, and oncolytic viruses can promote the activation of tumor immune cells, enhancing the collaborative effect.^[^ ^14^, ^15^ ^]^ Cryo‐immunization engineering was used to promote CTLs in changing the tumor microenvironment, improving the anti‐tumor effect.^[^ ^16^, ^17^ ^]^ Iron nanoparticles and biomineralized bacteria have been found to promote a shift from M2 to M1 macrophages. This shift enhances apoptosis and strengthens the anti‐tumor effect.^[^ ^18^, ^19^, ^20^, ^21^, ^22^ ^]^ Immunotherapy focused on the TME has exhibited clinical prospects.^[^ ^23^, ^24^, ^25^, ^26^, ^27^ ^]^ Intra‐tumor/Peri‐tumor chemotherapy is a novel treatment approach that targets the immune microenvironment within breast cancer cells. However, despite the overall ineffectiveness, ≈80% of patients undergo different levels of pain during treatment, specifically due to chemotherapy‐induced peripheral neuropathic pain (CIPNP) caused by Cisplatin (DDP).^[^ ^28^ ^]^ Chronic pain has been found to have an impact on the immune system, as well as on physical and mental health.^[^ ^29^, ^30^ ^]^ Consequently, it is crucial to create an anesthesia strategy that not only eases pain associated with chemotherapy but also as an immune modulator, enhancing the immune system and facilitating speedy recovery.

Opioids or local anesthetics are often used by doctors to manage chemotherapy‐related pain.^[^ ^31^, ^32^ ^]^ However, the immunosuppressive and toxic side effects greatly restrict the clinical applications.^[^ ^33^ ^]^ Esketamine is an N‐Methyl‐D‐aspartic acid (NMDA) receptor antagonist, widely used in the management of tumor‐related pain and depression.^[^ ^34^, ^35^, ^36^, ^37^ ^]^ Researchers discovered that Esketamine can also inhibit tumor growth by promoting necrosis, ferroptosis, and other mechanisms.^[^ ^38^, ^39^ ^]^ A study in lung adenocarcinoma found that Esketamine can promote apoptosis by activating the expression of CD69.^[^ ^40^ ^]^ Our study discovered that Esketamine has the ability to enhance the effectiveness of DDP as an immune modulator and improve the anti‐tumor efficacy in breast cancer.

Combination therapy faces several challenges in application, including rapid metabolism and difficulty in maintaining a consistent effective concentration. Increasing the dosage or frequency of chemotherapy drugs can worsen the toxic and side effects on the liver, kidney, and other metabolic organs.^[^ ^41^, ^42^ ^]^ A well‐designed system can enable targeted drug delivery, thereby enhancing drug concentration at the specific site. In order to improve the efficacy of combination therapy, it is crucial to prolong the duration of action, enhance the therapeutic effect, and reduce the side effects.^[^ ^43^, ^44^, ^45^ ^]^ The current drug delivery systems that are widely used include hydrogel systems,^[^ ^46^, ^47^ ^]^ nanoparticles,^[^ ^48^, ^49^ ^]^ micelles,^[^ ^50^, ^51^ ^]^ and liposomes.^[^ ^52^ ^]^ Injectable hydrogels were widely used in clinical practice for their excellent bio‐compatibility and degradability.^[^ ^53^ ^]^ Hydrogel, a polymer chain suspended in a watery substance, is created by cross‐linking using various processes. It is categorized into natural and synthetic hydrogels depending on its origin. Scientists use Poloxamer (P407) as a vehicle for administering chemotherapy drugs because of its temperature‐responsive characteristics. It can change into a liquid state at 4 °C or room temperature, making it easier to dispense and administer. As the temperature rises, it undergoes gelation and forms a semi‐solid gel. This enabled a continuous diffusion release and an extended period of drug encapsulation. Researchers widely used it in various administrations, such as transrectal injection, external eye administration, targeted injection, oral administration, nasal mucosal administration, and transdermal administration.^[^ ^54^ ^]^ By implementing local drug delivery, several advantages can be achieved, including improved drug targeting, reduced trauma from regular implants, and sustained release with minimal toxicity. By using P407 hydrogel‐loaded Doxorubicin, the researchers achieved a significant impediment to the growing progress in tumor local immune chemotherapy.^[^ ^55^ ^]^

**Figure 1:**
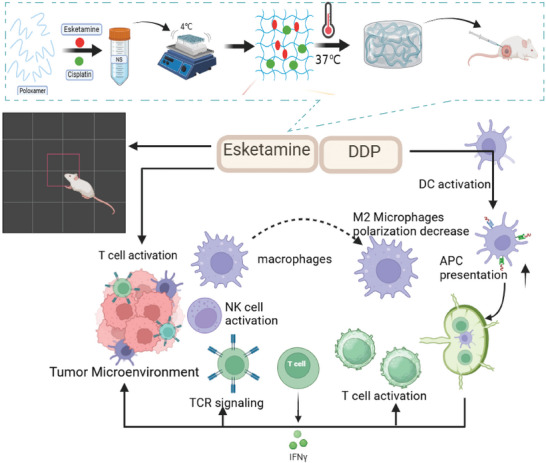
Schematic illustration of the PDEH. The diagram illustrates that Esketamine and DDP co‐load on P407 to create a hydrogel skeleton, and its aqueous solution can transform into a semi‐solid gel at 37 °C. The combined application of DDP has multiple benefits. It can induce pro‐inflammatory effects on DCs, T cells, and macrophages. This activation of DCs leads to enhanced presentation, resulting in the generation of numerous effector T cells in the lymph nodes. Ultimately, this leads to an increase in tumor‐infiltrating lymphocytes (TILs), which effectively kill tumor cells. Additionally, the TCR signaling pathway is upregulated and T cells continue to spread, ensuring a sustained immune response, and enhancing the efficacy. The tumor‐related anxiety and depression were also alleviated.

Preliminary studies have confirmed that Esketamine can enhance the anti‐tumor effect of DDP. We carried further research by constructing a hydrogel system of Esketamine combined with DDP co‐loaded in Poloxamer Hydrogel (PDEH). When comparing NS with PDEH, the findings indicated that PDEH can inhibit tumor growth and enhance the lifespan of mice. Flow cytometry revealed that PDEH activates the local immune response by enhancing the ability of mature DCs to present tumor‐associated antigens to CD8^+^ and CD4^+^ T cells. After being transported to the tumor site, T cells kill tumor cells through cytotoxicity mechanisms, such as interferon‐γ (IFN‐γ) release. Once the tumor cells are eliminated, they release a substantial number of tumor antigens. These antigens can be captured, processed, and presented by antigen‐presenting cells (APCs). Consequently, the induction of polyclonal T cells and decreased regulatory T cells (Tregs, CD4^+^CD25^+^FoxP3^+^), enhance the scope and strength of anti‐tumor immune responses. RNA sequencing confirmed the innate/adaptive immune related pathways, such as natural kill cell (NK) and T‐cell receptor (TCR) signaling pathway, while the hypoxia‐inducible factor‐1α (HIF‐1α) pathway down‐regulated and M2 macrophages decreased. In the PDEH group, we noticed a substantial inhibition of tumor growth and an extended lifespan. This study was the first to show that combining Esketamine with chemotherapy improves its effectiveness. Additionally, the results indicate that Esketamine can significantly reduce tumor‐related anxiety and depression. This innovative proposal suggests using Esketamine as an immune modulator to enhance the anti‐tumor effects of chemotherapy by improving the TME. This provides a new insight into the comprehensive painless treatment of breast cancer (**Figure** **1**).

## 2 Results

### 2.1 Characterization of PDEH

**Figure 2:**
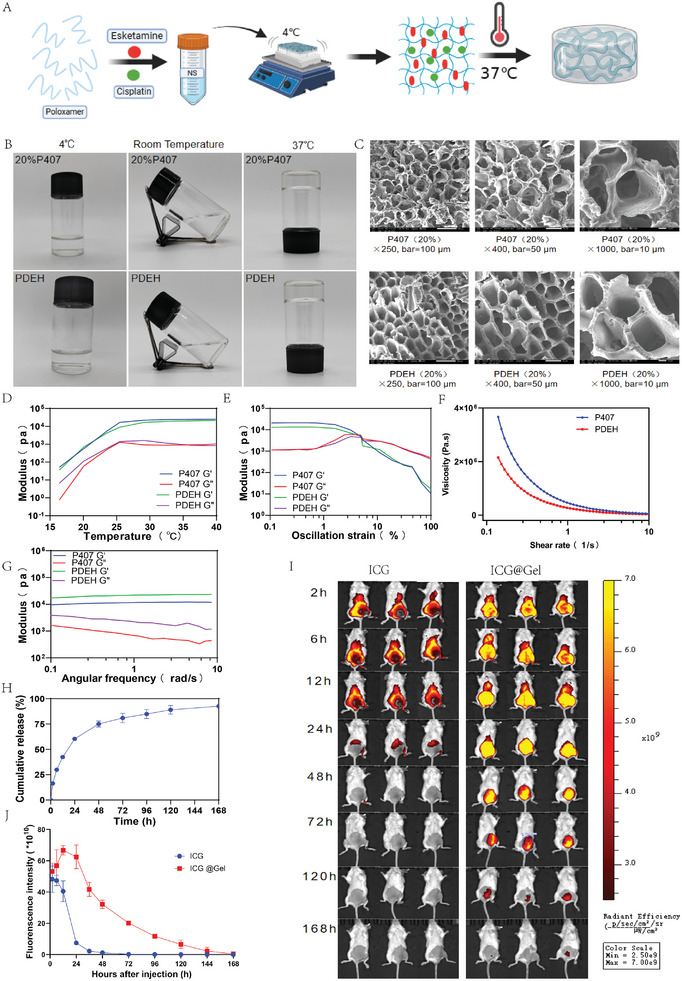
Characteristics of hydrogels P407 and PDEH. A) The process of PDEH; B) The formation of hydrogels at different temperatures; C) The 3D structure images scanning by SEM; D) Rheological analysis of temperature at 15–40 °C; E) Rheological analysis of oscillation behavior; F) Rheological analysis of shear; G) rheological analysis of angular frequency; H) The release of ICG hydrogel in vitro; I) The retention time (2, 6, 12, 24, 48, 72, 120, and 168 h) of ICG and ICG @Gel in vivo (n = 3); J) Corresponding quantitative analysis of the fluorescence intensity in ICG and ICG@Gel at 2, 6, 12, 24, 48, 72, 120, and 168 h in vivo, respectively (n = 3). The error bars represented mean ± SEM. (RT: Room Temperature).

Based on our previous study, it was confirmed that a 20% concentration of P407 was the most appropriate for the experiment.^[^ ^56^ ^]^ The formation of P407 and PDEH was investigated at different temperatures: 4 °C, room temperature, and 37 °C. Scanning Electron Microscope (SEM) and rotational rheometer were used for characterization detection. The release of drugs was studied using a small animal in vivo imaging system. We presented the preparation processes of P407 and PDEH (**Figure** **2**A). P407 and PDEH were liquid at 4 °C and room temperature but formed a semisolid gel at 37 °C (Figure 2B). SEM analysis showed that the 3D microstructure of P407 and PDEH did not change significantly (Figure 2C). Rheological properties are shown in Figure 2D–G. The shear analysis revealed that, as the viscosity of P407 and PDEH decreased, the shear became thinner, indicating good flow behavior (Figure 2D). At 37 °C, hydrogels exhibited solid‐like behavior, with G’ greater than G“ and a G”/G’ value close to 0. The behavior of PDEH was consistent with P407 (Figure 2E). The stabilities of P407 and PDEH were similar (Figure 2F), and the elastic behavior indicated good coherence of the hydrogels (Figure 2G). Overall, the characterizations of PDEH were not significantly different from P407. In vitro results showed the Indocyanine Green (ICG) release was 44.78% at 24 h, 62.1% at 48 h, and almost 92.3% at 168 h (Figure 2H). In vivo, we compared the subcutaneous half‐life of ICG and Indocyanine green poloxamer hydrogel (ICG@Gel), and found that ICG was rapidly cleared by day 2 (Figure 2I, left picture), while ICG@Gel remained for almost 7 days (Figure 2I, right picture), showing that hydrogel could prolong the half‐life, which increased the subcutaneous retention of ICG. Fluorescence intensity was measured at 2, 6, 12, 24, 48, 72, 120, and 168 h, showing significant differences between the ICG and ICG@Gel groups (Figure 2J). It is speculated that the co‐loaded hydrogel can rapidly release drugs within 24 h, exerting a quick anti‐tumor effect, followed by a continuous release over 7 days.

### 2.2 PDEH Inhibited the Tumor Growth

**Figure 3:**
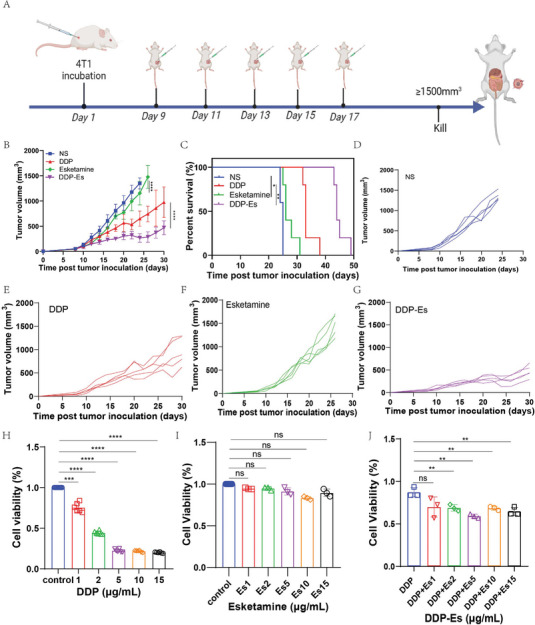
Anti‐tumor efficacy and safety of DDP‐Es. A) Schematic diagram of drug administration for treating the tumors; B) Growth curves represent the average tumor volumes of each group (n = 5); C) Tumor survival time in each group; D) Tumor growth of each mouse in NS groups (n = 5); E) Tumor growth of each mouse in DDP groups (n = 5); F) Tumor growth of each mouse in Esketamine groups (n = 5); G) Tumor growth of each mouse in DDP‐Es groups (n = 5); H) Cytotoxicity of different concentrations of DDP on 4T1 cells (n = 5); I) Cytotoxicity of different concentrations of Esketamine on 4T1 cells (n = 3); J) Cytotoxicity of DDP‐Es in 4T1 cells (n = 3). The error bars represented mean ± SEM. P‐values were calculated by two‐way ANOVA and Tukey post‐test and correction in Figure B or two‐tailed unpaired Student's t‐tests in Figure H–J. ns represented *p* > 0.05, ^**^ represented *p* < 0.01, ^***^ represented *p* < 0.001, ^****^ represented *p* < 0.0001.

The anti‐tumor effect of DDP‐Esketamine (DDP‐Es) was verified using the 4T1 mouse breast cancer model. Once the tumor reached a size of ≈80–100mm^3^, mice were randomly divided into four groups: NS group, Esketamine (Es) group, DDP group, and DDP‐Es group. The treatment is shown in **Figure** **3**A. The DDP‐Es group exhibited a significantly slower tumor growth rate compared to the DDP and NS groups (P < 0.0001) (Figure 3B) while the survival time extended in the DDP‐Es group compared to the DDP group (P = 0.0020) (Figure 3C). These suggest that Esketamine can synergistically inhibit tumor growth with DDP. The tumor growth values for each group, as depicted in Figure 3D–G, correlated well with Figure 3B. The cytotoxicity of 4T1 cells positively correlated with the dose of DDP at different concentrations (Figure 3H). Additionally, Esketamine exhibited some cytotoxicity on 4T1 cells at different concentrations although there was no significant difference (Figure 3I). Furthermore, Cell Counting Kit‐8 (CCK‐8) analysis also revealed that Esketamine enhanced the cytotoxicity of DDP on 4T1 cells (P < 0.01) (Figure 3J).

**Figure 4:**
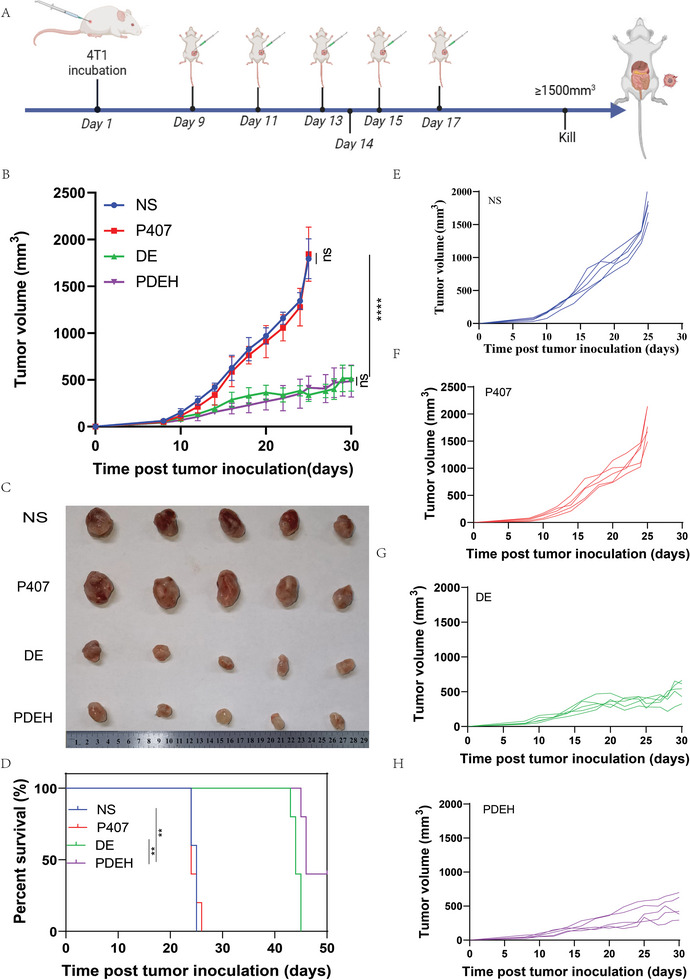
Evaluation of the efficacy and safety of PDEH in Balb/c mice. A) Schematic diagram of drug administration in treating the tumors; B) Growth curves represent the average tumor volumes of each group (n = 5); C) The tumors excised from each group; D) Tumor survival time of each group (n = 5); E) Tumor growth of each mouse in NS group (n = 5); F) Tumor growth of each mouse in P407 group (n = 5); G) Tumor growth of each mouse in DE group (n = 5); H) Tumor growth of each mouse in PDEH group (n = 5). The error bars represented mean ± SEM. P‐values were calculated by two‐way ANOVA and Tukey post‐test and correction in Figure B or log‐rank (Mantel–Cox) test in Figure D. ns represented *p* > 0.05, ^****^ represented *p* < 0.0001.

Four groups were established for further experimental study: NS group, P407 group, DDP‐Esketamine (DE) group, and PDEH group. Based on the previous findings, we suggest that combining Esketamine with DDP co‐loaded in P407 hydrogel can enhance the anti‐tumor effect. The protocol for setting up the groups remained unchanged, and the procedure is outlined in **Figure** **4**A. P407 did not exhibit any anti‐tumor effect compared to the NS group (P = 0.1260), indicating that the hydrogel was non‐toxic, which is consistent with the CCK‐8 results in Figure 7A,C,D. We suspect that the lack of a statistically significant difference in tumor size between the DE and PDEH groups (P = 0.3160) is related to the residual hydrogel (Figure 4B). The excised tumors in all four groups were consistent with the tumor growth and survival time (Figure 4C). The PDEH group had a significantly longer lifespan compared to the DE group (P < 0.01), and it was nearly twice as long as the NS group with a significant difference (P = 0.0084) (Figure 4D). Diagrams in Figure 4E–H illustrate the individual growth values of tumor growth in the NS, P407, DE, and PDEH groups. These results support the findings in Figure 4B–D.

### 2.3 PDEH Activated the Anti‐Tumor Response Through TDLNs

**Figure 5:**
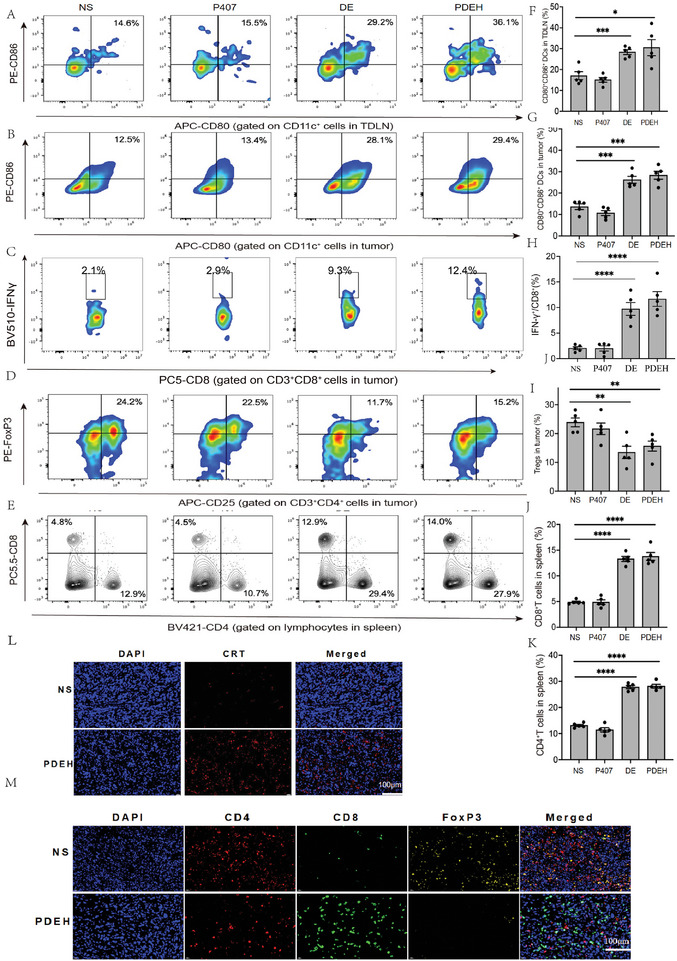
The anti‐Tumor response through TDLN. A) Representative flow cytometry of mDCs in TDLN; B) Representative flow cytometry of mDCs in tumor; C) Representative flow cytometry of CD8^+^ T cells in tumor; D) Representative flow cytometry of Tregs in tumor; E) Representative flow cytometry of T cells in spleen; F) Statistical graph of mDCs in TDLN; G) Statistical graph of CD8^+^ in tumor; H) Statistical graph of mDCs in tumor; I) Statistical graph of Tregs in tumor; J) Statistical graph CD8^+^ lymphocytes in spleen; K) Statistical graph CD4^+^ T cells in spleen; L) Immunofluorescence of CRT in tumor tissue (bar = 100 µm); M) Immunofluorescence of Tregs (FoxP3) in tumor tissue (bar = 100 µm). The error bars represented mean ± SEM. P‐values were calculated by two‐tailed unpaired Student's t‐tests. ns represented *p* > 0.05, ^*^ represented *p* < 0.05, ^**^ represented *p* < 0.01, ^***^ represented *p* < 0.001, ^****^ represented *p* < 0.0001.

After confirming the suppressive effect of PDEH on tumors, we examined the immune‐related cells in tumor‐draining lymph nodes (TDLN) and tumors. In TDLN, the proportion of activated DCs in the PDEH group increased from 14.6% in the NS group to 36.1% (**Figure** **5**A). The PDEH group also showed a significant increase in mDCs (CD11c^+^CD80^+^CD86^+^) cells compared to the NS group (Figure 5F), with a statistically significant difference (P < 0.05). mDCs are efficient at delivering antigens to T cells, and the content of mDCs in tumors in the PDEH group increased to 29.4% compared to 12.5% in the NS group, representing a 2.4‐fold increase (Figure 5B) with statistical significance (P < 0.05). However, there was no statistically significant difference between the DE and PDEH groups (P < 0.99) (Figure 5G). The presence of mDCs in TDLN can stimulate T cells in tumors, leading to an influx of immune cells in the tumor through peripheral circulation. Our analysis mainly focused on the changes in T cells in the tumor and spleen. An increase in tumor infiltrating lymphocytes (TILs) is indicative of a favorable immune response. PDEH significantly increased the number of CD8^+^ TILs. IFN‐γ is a cytokine secreted by CD8^+^ T cells, which plays a role in killing tumor cells after exposure to pre‐inoculated antigens. The proportion of IFN‐γ expressing CD8^+^ T cells increased in all treatment groups, with the PDEH group having the highest proportion at 12.4%, a sixfold increase compared to the NS group (Figure 5C). This difference was statistically significant compared to the NS group (P < 0.0001) (Figure 5H). The proportion of Tregs in the PDEH group experienced a substantial decrease of almost 9.0%. This is in stark contrast to the significantly higher 24.2% observed in the NS group (Figure 5D,I). Additionally, we noticed an increase in the proportion of CD4^+^ T cells and CD8^+^ T cells in the spleen. CD4^+^ T cells increased from 12.9% to 27.9%, while CD8^+^ T cells increased from 4.8 to 14% between the NS and PDEH groups (Figure 5E). The PDEH group exhibited a significant increase in CD8^+^ T cells (P < 0.001) and CD4^+^ T cells (P < 0.001) in the spleen compared to the NS group (Figure 5J,K). These results suggest a prolonged immune response to PDEH treatment. Immunofluorescence images of PDEH showed a significant improvement in calreticulin (CRT), indicating the induction of immunogenic cell death and inhibition of tumor growth (Figure 5L). Tregs ( FoxP3^+^) images displayed an increase in the NS group, while CD8^+^ was significantly higher in the PDEH group (Figure 5M). These findings confirm that PDEH works by inhibiting Tregs and activating TILs in the microenvironment, which aligns with previous results (Figure 5D,I).

### 2.4 PDEH Activated the Innate/Adaptive Immune Response

Transcriptome sequencing was conducted on tumors in NS and PDEH, and the data was analyzed for immune‐related pathways. ≈632 differential genes were identified, including 360 up‐regulated genes and 272 down‐regulated genes (Figure S1A, Supporting Information). The analysis revealed various components of immune responses, including biological processes, cellular components, and molecular functions (Figure S1B, Supporting Information). Gene Ontology (GO) enrichment analysis of the immune‐related pathways mainly focused on immune processes and immune responses (Figure S1C, Supporting Information). The Gene Set Enrichment Analysis (GSEA) expression analysis was then performed on the immune‐related pathways (Figure S1D–G, Supporting Information).

The pathway regulated by HIF‐1α, which plays a role in regulating multiple signaling pathways in tumor progression and cell apoptosis and autophagy, was significantly inhibited in the PDEH group (Figure S1D, Supporting Information). This inhibition was accompanied by increased proliferation and TILs (Figure S1E, Supporting Information), which played a crucial role in cancer cell death. Additionally, the TCR pathway was also activated (Figure S1F, Supporting Information). TCR can recognize antigens and up‐regulate pathways such as Mitogen‐Activated Protein Kinase (MAPK), Protein Kinase C (PKC), and calcium ions, ultimately leading to T cell activation. Furthermore, enhanced cytotoxicity mediated by NK cells was observed (Figure S1G, Supporting Information). NK cells exhibit unique anti‐tumor effects, including cytotoxicity, cytokines production, and immune memory functions, making them key players in the innate and adaptive immune response systems.

The immune‐related pathways analyzed by Kyoto Encyclopedia of Genes and Genomes (KEGG) were depicted in a bubble diagram (Figure S1H, Supporting Information), which demonstrated consistency with the aforementioned research results. Finally, CYBERSORT analysis revealed a significant decrease in M2 macrophages and a substantial increase in CD8^+^ T cells in the PDEH group (P = 0.043) (Figure S1I, Supporting Information). In conclusion, NK cells, T cells, and their associated pathways were activated in the immune‐related anti‐tumor process of PDEH, while M2 macrophages and HIF‐1α decreased.

### 2.5 PDEH Improved the Anxiety and Depression in 4T1 Tumor Mice

**Figure 6:**
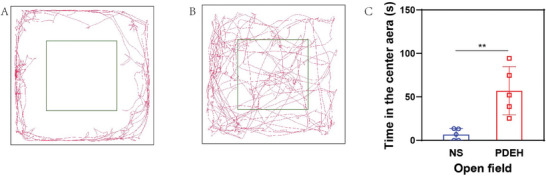
Open field experiment for the anxiety and depression detection in 4T1 tumor mice. A) Action trajectory of NS group; B) Action trajectory of PDEH group; C) Statistical analysis of residence time in a central area between NS and PDEH group (n = 5). The error bars represented mean ± SEM. P‐values were calculated by two‐tailed unpaired Student's t‐tests in Figure C. ^**^ represented *p* < 0.01.

The movement trajectory and the residence time entered the central area were measured. Near‐infrared movement trajectory was shown in the NS group (**Figure** **6**A), while the trajectories in the central region significantly increased in the PDEH group (Figure 6B). The dwell time entering the central area in PDEH was significantly higher than the NS group (P = 0.0043), suggesting that PDEH can improve anxiety and depression significantly (Figure 6C). Based on these findings, we propose that PDEH holds promising clinical application value.

### 2.6 Biosafety Assessment

**Figure 7:**
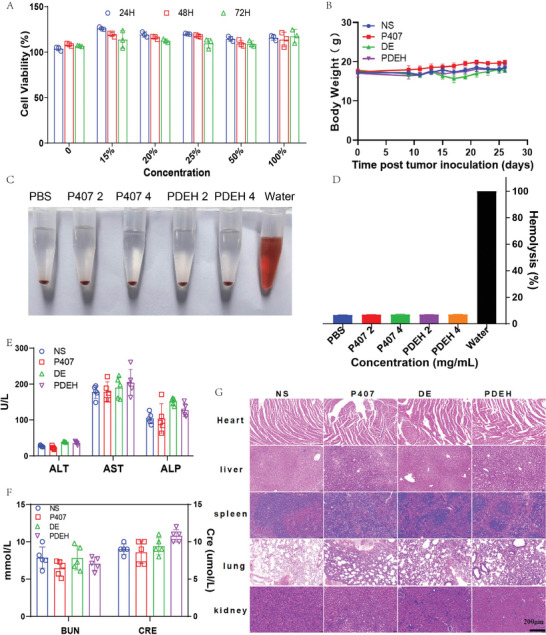
Biosafety assessment of PDEH. A) Cytotoxic effects of varying hydrogel extract concentrations on HUEVC at 24, 48, and 72 h; B) Weight changes of mice in the groups during treatment (n = 5); C) Hemolysis test of different concentrations of hydrogels carried by RBCs; D) Quantitative analysis of the Hemolysis test (n = 3); E) Liver function indices (n = 5); F) Kidney function indices (n = 5); G) H&E staining of Important organs of each group (Heart, liver, spleen, lung, and kidney) (n = 5, bar = 200 µm). The error bars represented mean ± SEM. P‐values were calculated by two‐way ANOVA in Figure B and two‐tailed unpaired Student's t‐tests in Figures D–F.

The safety evaluation of hydrogels involved CCK‐8 on Human Umbilical Vein Endothelial Cells (HUEVC) and the hemolysis test. We co‐cultured different concentrations of hydrogel extracts with HUEVC for 24, 48, and 72 h (**Figure** **7**A). CCK‐8 demonstrated that the survival rate of all cells exceeded 95%, showing excellent biological safety of the hydrogel with no significant cytotoxicity. We paid extra attention to the potential security of PDEH during the research. Hemolysis was not observed in the P407 and PDEH groups when compared to water in red blood cell (RBCs) suspensions (Figure 7C,D). This confirms that the P407 hydrogel was suitable to be a drug carrier. When the treatment of 4T1 tumor in Balb/c mice was examined, it was observed that the body weight of mice in the PDEH group remained normal compared to other groups without abnormal fluctuations (Figure 7B). Liver and kidney function were tested in mice at the end of the study. The levels of aspartate aminotransferase (AST), alanine aminotransferase (ALT), alkaline phosphatase (ALP), blood urea nitrogen (BUN), and creatinine (CRE) in each group showed a basic consistency and were within the normal range (Figure 7E,F). In addition, the Hematoxylin‐Eosin (H&E) staining images of major organs (heart, liver, spleen, lung, kidney) showed there was no visible damage in any of the groups (Figure 7G). The results showed that PDEH exerted significant local immune activation without systemic side effects, and did not damage liver and kidney functions. Overall, the study demonstrated that the thermo‐sensitive hydrogel system PDEH was biologically safe.

## 3 Discussion

The effectiveness and pain‐related issues of chemotherapy have a significant impact on the prognosis of the breast tumor. Unfortunately, both malignant tumor cells and normal immune cells are destroyed due to the imprecision of chemotherapy. Immune cells play a critical role in suppressing the growth of tumors. As a result, targeting the microenvironment with immunotherapy has emerged as a promising treatment for improving efficacy.^[^ ^12^ ^]^ However, the pre‐existing T cells found in the microenvironment influenced the effects of immunotherapy. The limited T cells and mDCs, coupled with an abundance of immunosuppressive cells, hindered the successful presentation of antigens and limited the functionality of T cells, ultimately leading to ineffective immunotherapy.^[^ ^57^ ^]^ 4T1 breast cancer was a triple‐negative tumor with abundant immunosuppressive cells and tiny T cells. Regulating the immunosuppressive microenvironment, and inducing T‐cell infiltration to improve the immune response was crucial.^[^ ^17^ ^]^ However, there were still other problems that cannot be ignored. ≈80% of patients experienced different degrees of pain, and chronic pain has a devasting impact on the immune system, particularly on immune T cells. Increased pain levels can lead to increased inflammatory factors, negatively affecting immune cells and their function. This can significantly affect the prognosis of individuals with tumors.^[^ ^58^, ^59^ ^]^ Thus, it is essential to develop an appropriate anesthesia strategy that incorporates a potential modulator. The strategy mentioned aims to address multiple issues related to cancer treatment. It focuses on alleviating pain, enhancing immune cell activity, and improving the effectiveness of DDP against tumors.

This study proved that the simultaneous delivery of Esketamine and DDP in P407 hydrogel consistently achieved a concentrated presence at the tumor site. Various benefits are offered by P407 hydrogels, making them widely used.^[^ ^60^ ^]^ First, it is typically administered via peri‐tumoral injection. This method effectively enhances drug concentration at the intended site, while simultaneously minimizing systemic toxicity. Second, the preparation that involved mixing hydrogels with other drugs was easy to go. Crosslinking agents, organic solvents, and intricate chemical synthesis steps were not required. Scientists used P407 hydrogel to deliver Dorobicin for localized tumor immunotherapy, resulting in slowed tumor growth and a significant anti‐tumor effect.^[^ ^32^ ^]^ PF127 hydrogel loaded ropivacaine and DDP was used for intra‐tumor chemotherapy in breast cancer, indicating a potential effect on inhibiting tumor growth by enhancing CD8^+^ T cells in the microenvironment.^[^ ^61^ ^]^ By incorporating imiquimod and ropivacaine into a hydrogel, researchers were able to prevent tumor recurrence by enhancing CD8^+^ T cells within the tumor.^[^ ^62^ ^]^ Esketamine was well‐utilized in tumor treatments, providing adequate pain relief. According to a study conducted on gastric cancer, ketamine has been shown to possess anti‐tumor properties by promoting apoptosis and modulating the PI3K/Akt/mTOR pathway.^[^ ^63^ ^]^ The activation of NK cells by Esketamine was found to have an anti‐tumor immune effect in studies conducted on pancreatic cancer and lung adenocarcinoma.^[^ ^64^ ^]^ In our previous study, we made a fascinating discovery regarding the potential anti‐tumor properties of Esketamine when combined with DDP. We were thrilled to observe that the combination of Esketamine and DDP, both co‐loaded in P407 hydrogel, effectively suppressed the growth of 4T1 tumors. The findings of the study revealed several key observations: 1) there was a noticeable increase in antigen accumulation in the lymph nodes, along with a higher proportion of activated DCs, as depicted in Figure 5A,B. 2) Additionally, there was a threefold increase in the proportion of tumor‐infiltrating specific IFN‐γ cytokines, shown in Figure 5C,H. 3) Furthermore, there was a significant presence of toxic T cells in the spleen, as illustrated in Figure 5E,J,K. The successful work of PDEH relies on the production of activated cells, which mediate the cellular immune response. PDEH initiates the immune system through the process of antigen presentation conducted by mDCs. Additionally, DCs produce cytokines that aid in the production and expansion of tumor‐specific CD8^+^ and CD4^+^ T cells. These T cells then migrate to the tumor site and eliminate tumor cells through cytotoxicity and cytokine secretion, such as IFN‐γ. As a result, the killed tumor cells release tumor antigens, which are captured, processed, and presented by APCs. This leads to the induction of polyclonal T‐cell responses, enhancing the breadth and intensity of anti‐tumor immune responses. However, the upregulated inhibitory molecules and cells like Tregs and M2 macrophages lead to an immune‐resistance.^[^ ^65^, ^66^ ^]^ In our study, Tregs and M2 microphages decreased significantly in PDEH (Figure 5D,I,M; Figure S1I, Supporting Information).

There are some limitations and plans for our work. It is crucial to have a comprehensive understanding of the underlying mechanisms when verifying the broad‐spectrum activation. To explain this, we performed RNA‐sequencing, as shown in Figure S1 (Supporting Information), and the reduction of M2 macrophages and increased infiltration of TILs were confirmed. This regulates the immune microenvironment and has anti‐tumor effects. The upregulated pathways of NK and TCR (Figure S1F,G, Supporting Information), as well as the downregulated HIF‐1α signaling pathway (Figure S1I, Supporting Information), were associated with the anti‐tumor effect of PDEH. However, it is important to note that key proteins integral to these pathways play a crucial role in enhancing the robustness of our proposed mechanistic framework. In future investigations, we plan to conduct western blot experiments, including HIF‐1α and others, to confirm any changes in the expression levels of important proteins. Additionally, we are dedicated to carrying out single‐cell sequencing to investigate the role of immune cells.

The prevalence of cancer‐related depression (CRD) in cancer patients is estimated to be ≈65%−78%.^[^ ^67^ ^]^ Studies have shown that psychological disorders can contribute to the progression of tumors.^[^ ^68^ ^]^ The Duke Bing teams have discovered that chemotherapy‐related peripheral neuropathic pain can worsen psychological disorders. This is where the role of kisspeptin/GPR54 signaling becomes important. GPR54 promotes T cell exhaustion in the tumor microenvironment and facilitates immune escape.^[^ ^69^ ^]^ Numerous investigations have demonstrated the positive effects of Esketamine in reducing anxiety and depression. The mechanism behind this involves the function of Th17 cells.^[^ ^70^ ^]^

To evaluate the emotional behavior of mice, the Open Field test measured parameters, such as the horizontal action trajectory, total action distance, and central Grid residence time were included. Our study found that PDEH significantly increased the residence time in the central area, suggesting its effectiveness in alleviating anxiety and depression in mice. The underlying mechanism is likely to be associated with Th17 cells, as confirmed by transcriptome RNA sequencing (Figure S1H, Supporting Information), which is consistent with previous findings.^[^ ^70^ ^]^

## 4 Conclusion

Overall, this study presents the development of a unique hydrogel system that contains both Esketamine and DDP. Using a combination strategy targets the local immune system, effectively reducing toxic effects and enhancing drug utilization, ultimately improving the inhibition of tumor growth. The activated DCs lead to a more efficient activation of T‐cell immune responses. Additionally, it reduces immune‐depressive Tregs in the microenvironment. The hydrogel system tested in 4T1 mouse models has shown both anti‐tumor effects and safety, indicating its potential for clinical translation. Furthermore, Esketamine loaded in P407 can also improve anxiety and depression caused by tumors, offering a workable combination strategy for painless tumor immunotherapy.

## 5 Experimental Section

### Materials and Antibodies

The company Beijing Bio Legend supplied us with various antibodies including Anti‐mouse CD11c‐FITC, Anti‐mouse CD4‐BV421, Anti‐mouse CD80‐APC, Anti‐mouse CD86‐PE, Anti‐mouse IFN‐γ‐PE, Anti‐mouse FoxP3‐PE, Anti‐mouse CD3‐FITC, Anti‐mouse CD11b‐FITC, Anti‐mouse CD62L‐APC, Anti‐mouse CD8‐Percp‐Cy5.5, Anti‐mouse CD44‐PE, Anti‐mouse CD206‐APC, Anti‐mouse CD25‐APC, Anti‐mouse F4‐80‐PCT, and Anti‐mouse CD49b‐FITC. The Cytonix/Cytoperm fixation/breaking membrane kit was purchased from BD Company in the United States, and Type IV collagenase and poloxamer powder from Sigma–Aldrich Company in the United States. Additionally, ICG was obtained from Shanghai Aladdin Co. Ltd, Esketamine from Jiangsu Heng Rui Pharmaceutical Co., Ltd, and DDP from Shanghai Aladdin Biochemical Technology Co. Ltd.

### Mice and Cell Lines

5‐6‐week‐old Balb/c mice were obtained from Shanghai Sippr‐BK laboratory animal Co.Ltd. in Shanghai, China. The mice were housed in the specific pathogen‐free (SPF) Laboratory Animal Center at the Affiliated Nanjing Drum Tower Hospital of Nanjing University Medical School in Nanjing, China. They were kept under controlled conditions of temperature (68–79 °F), humidity (30–70%), and a 12‐h light/dark cycle (lights on from 6 am to 6 pm). The mice had ad libitum access to feed and water. All animal experimental procedures were approved by the Laboratory Animal Care and Use Committee of the Affiliated Nanjing Drum Tower Hospital of Nanjing University Medical School.

The 4T1 mouse breast cancer cell line was purchased from the Cell Bank of the Chinese Academy of Sciences in Shanghai, China, while the HUEVC cell line was obtained from the Obstetrics and Gynecology Department of Drum Tower Hospital. Both cell lines were cultured in an RPMI 1640 medium supplemented with 10% fetal bovine serum (FBS) at 37 °C in a 5% CO2 atmosphere.

### Preparation and Characterization of Hydrogels

A 20% P407 was prepared by dissolving 200 mg of poloxamer powder in either PBS or ddH2O and stirring at 4 °C until the solution became clear. To create a 20% PDEH hydrogel, 200 mg of poloxamer powder, 1 mg of DDP, and 10 mg of Esketamine were dissolved in a 50 ml centrifuge tube. The solution was gently mixed and stirred at 4 °C until it became clear. Then the hydrogels were put in a −80 °C refrigerator for 24 h and dried in a low‐temperature vacuum. Using scanning electron microscopy (SEM: JSM‐6330F, JEOL, Japan) to analyze the morphology of hydrogels.

### Rheological Properties

The rheological characterization of hydrogels was carried out using a strain‐controlled shear rheometer (MCR 302; Anton‐Paar, Austria). The rheological properties of the hydrogels were measured, such as the storage modulus (G’) and loss modulus (G’’), at a constant frequency of 1.0 Hz and a strain amplitude of 1%. The temperature was gradually increased from 15 to 40 °C at a heating rate of 1 °C min^−1^. At a temperature of 37 °C, the oscillation strain, frequency dependence, and shear behavior were measured. All data were recorded and analyzed using parallel plates with a gap size of 0.6 mm and a geometry of 40 mm.

### Hydrogel Biosafety

A mixture of 1 mL of P407 and 10 mL of cell culture medium was gently shaken in a temperature oscillator at 37 °C for 24 h, resulting in a complete hydrogel extract. The extract was then diluted to different percentages (15, 20, 25, 50, and 100%) using the culture medium. It was co‐incubated with HUEVC for 24, 48, and 72 h, respectively. The cell survival rate was analyzed using CCK‐8.

### Hemolysis Test

The hemolysis assay was conducted using mice RBCs as the model cells. The RBCs were isolated and washed with PBS five times at 3000 rpm for 5 min. Subsequently, different concentrations of P407 and PDEH were added to the RBC suspensions and incubated for 3 h. The hemoglobin released in the supernatant was determined by measuring its absorbance at 540 nm. The degree of hemolysis was calculated relative to 100% hemolysis, which was treated with ultra‐pure water.

### Drug Release of Hydrogel

In order to address the challenges of detecting Esketamine, it is hypothesized that ICG could possess drug release properties. An experiment was conducted in which 200 µL of ICG@Gel was placed in the upper chamber of the Trans‐well board. 1 mL of PBS (pH 7.4) was added and incubated in the solution for various time intervals: 2, 6, 12, 24, 48, 72, 96, 120, and 168 h for in vitro detection. Additionally, ICG and ICG@Gel were injected subcutaneously in Balb/c tumor mice to investigate their retention time in vivo. Using a live small animal imaging platform, near‐infrared fluorescence images were immediately detected, as well as at 2, 6, 12, 24, 48, 72, 96, 120, and 168 h after injection. By measuring the ICG fluorescence values, the ICG release curve was able to calculate.

### Animal Experiment

Mouse breast cancer cells (4T1) were injected subcutaneously into the left lower abdomen of Balb/c mice at a concentration of 2 × 10^5^ cells per mouse on day 1. After ≈8 days, when the tumor had grown to nearly 80–100 mm^3^, the mice into four groups was randomly divided for the first experiment: NS group, DDP group (0.4 mg mL^−1^ of final concentration), Esketamine group (4 mg mL^−1^ of final concentration), and DDP‐Es group (0.4 mg mL^−1^ DDP and 4 mg mL^−1^ Esketamine of final concentration), with a total volume of 100 uL per mouse. Treatment began on day 9 and was administered once every other day for five times. Tumor volume was calculated using the formula V (mm^3^) = 0.5 × DL × DS^2^. Tumor sizes and body weights were measured every two days. Euthanasia was conducted when the tumors reached a volume of 1500 mm^3^.

In the subsequent study, The mice into four groups were categorized: NS group, P407 group (20 mg mL^−1^ of final concentration), DE group (0.4 mg mL^−1^ DDP and 4 mg mL^−1^ Esketamine of final concentration), and PDEH group (1 mL of the drug‐loaded hydrogels contains P407 20 mg, DDP 1 mg, Esketamine 10 mg of final concentration) were prepared. NS and DE groups were administered every other day (D9, D11, D13, D15, D17) for five times, while the P407 and PDEH groups were conducted on D9 and D14. Tumor sizes and body weights were measured every two days. On D22, the hearts, livers, lungs, and kidneys were excised for HE staining and immunofluorescence. Additionally, spleens, lymph nodes, and tumors were collected for Flow Cytometry and RNA sequencing.

### Flow Cytometry

The antibody 1:100 was diluted and the True‐Nuclear transcription factor buffer was set from Bio Legend to detect IFN‐γ and FoxP3 intracellular staining. After euthanizing the mice, tumor tissues, TDLN, and spleens were collected. To obtain single‐cell suspensions, filtered, and suspended the TDLN and spleen cells were mechanically scrubbed in NS at a concentration of 0.5‐1 × 10^6^ cells ml^−1^. The tumors were chopped into small pieces, then incubated with type IV collagenase (1 mg ml^−1^, Sigma, USA) at 37 °C for 3–4 h. The resulting cell suspension was filtered and suspended in NS at a concentration of 0.5‐1 × 10^6^ cells ml^−1^. All the samples were placed in pre‐cooled NS and stained with specific antibodies for 20 min in the dark at 4 °C, once most antigens had been expressed on the cell membranes. After staining, the samples were washed before analysis. Flow Cytometry was used for detection and FlowJo for data analysis.

### RNA Sequencing

The mice and swiftly excised tumors were euthanized in NS and PDEH for RNA sequencing (Berry Genetics, Beijing, China). The DESeq2 Bio conductor kit was used for gene expression quantification and differential expression analysis, and GOSeq (v1.34.1) was used. Functions related to biological processes were analyzed, and the KEGG database was used for enrichment analysis (http://en.wikipedia.org/wiki/KEGG). The CYBERSOT software was used to analyze the immune cellular infiltration in tumors.

### Immunofluorescence Staining

The tumors were surgically removed and treated with 4% paraformaldehyde for 4–6 h. Subsequently, the tissues were embedded in paraffin using a tissue embedding machine and then sliced into 10 µm sections using a cryostat. Following three washes with PBS, the frozen sections were blocked with 3% bovine serum albumin (BSA) at a temperature of 37 °C. The slides were then incubated overnight with a primary antibody at 4 °C, followed by a 1‐h incubation with a fluorescent secondary antibody and diamidino‐phenyl‐indole (DAPI) at 37 °C. Finally, the slides were sealed with 50% glycerol, and fluorescence images were captured using a confocal microscope (Leica, German).

### Behavior Assessment

The mice treated were placed with NS and PDEH in an open field of 50cm*50 cm for a duration of 5 min each. The movement trajectories were analyzed by dividing the field into central and peripheral areas. The mice traveled into the central area and were recorded and analyzed a number of times as a measure of anxiety and depression evaluation.

### Statistical Analysis

Unless otherwise stated, biological replicates were performed. All experiments were performed with a minimum of n = 3 per group. The analysis of comparative data between the two groups was conducted using a two‐sided Student's t test. Comparisons between three or more groups of data were analyzed using a one‐way or two‐way ANOVA. The survival benefit was determined by the Log‐rank (Mantel‐Cox) test. All statistical analyses and statistical charts were completed by Graph pad Prism 8.0.1 software. The mean ± standard deviation (mean ± SD) or mean ± standard error (mean ± SEM) expressed the data. ^*^ *p* < 0.05, ^**^ *p* < 0.01, ^***^ *p* < 0.001 and ^****^ *p* < 0.0001 were considered statistically significant.

## Conflict of Interest

The authors declare no conflict of interest.

## Supporting information

Supporting Information

## Data Availability

The data that support the findings of this study are available on request from the corresponding author. The data are not publicly available due to privacy or ethical restrictions.
