# Supplementary material for: An Immune‐Enhancing Injectable Hydrogel Loaded with Esketamine and DDP Promotes Painless Immunochemotherapy to Inhibit Breast Cancer Growth
Source: Adv Healthc Mater. 2024 Aug 9;13(29):2401373. doi: 10.1002/adhm.202401373 (PMC11582503; doi:10.1002/adhm.202401373)
Supplement: Supplementary file 1 — Supporting Information [file ADHM-13-0-s001.docx]

**Supporting information**


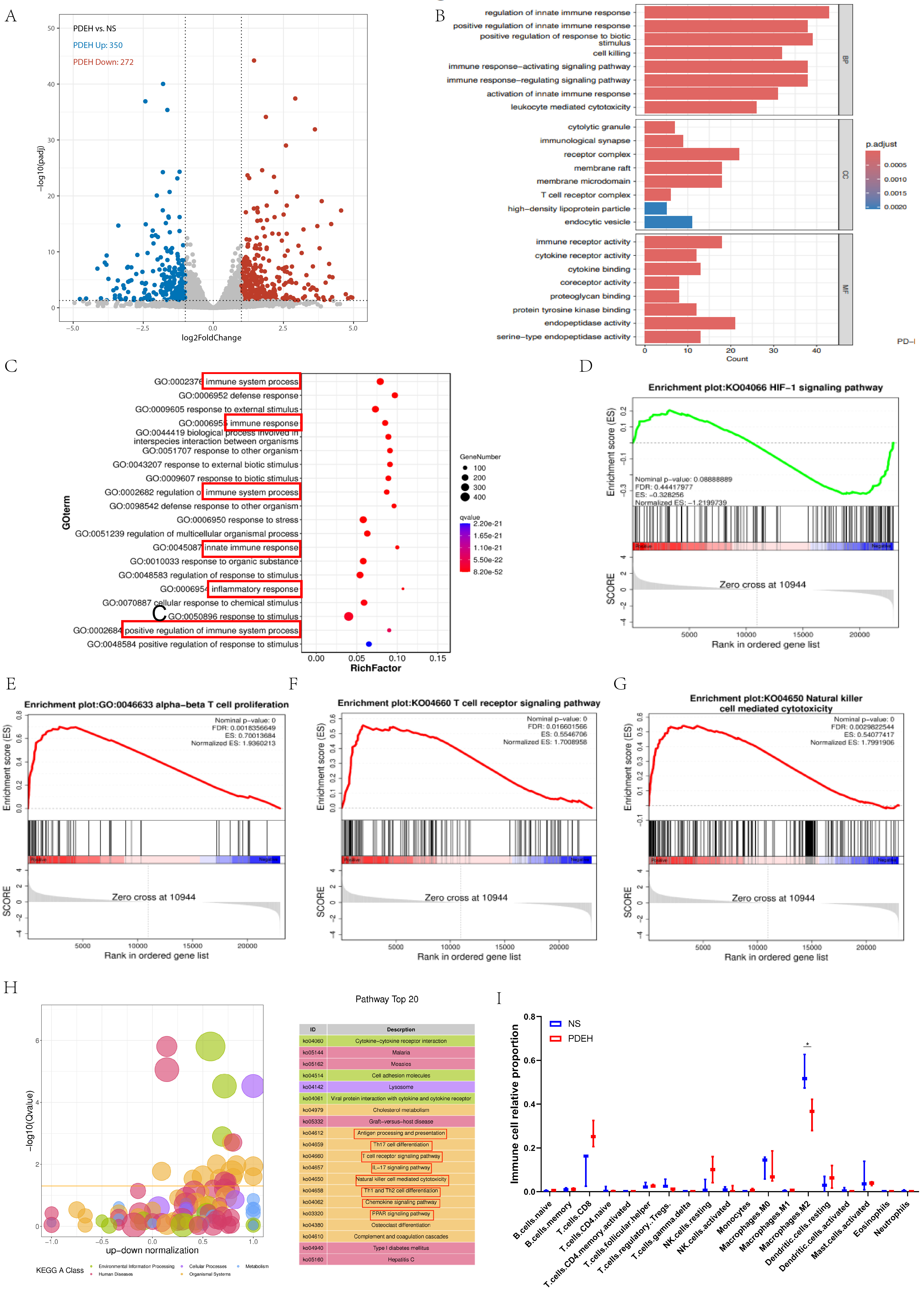


**Figure S1.** Immune related analysis in PDEH. A: Differential gene expression in NS group and PDEH group (volcano plot); B: immune responses of biology process, cellular component and molecular function; C: Immune-related signaling pathways by GO enrichment analysis; D: GSEA expression analysis of HIF-1α signaling pathway; E: GSEA expression analysis of proliferation and infiltration of T cells; F: GSEA expression analysis of T cell receptor signaling pathway; G: GSEA expression analysis of NK cell signaling; H: Bubble diagram of immune-related signaling pathways from KEGG enrichment analysis; I: CYBERSORT analysis of infiltrated immune cells. The error bars represented mean ± SEM. P-values were calculated by two-tailed unpaired Student’s t-tests Figure I. * represented p<0.05.
